# CD20-Negative Large B-Cell Lymphomas: The Diagnostic Challenge of Tumors with Downregulation of Mature B-Cell Marker Expression

**DOI:** 10.3390/ijms26167843

**Published:** 2025-08-14

**Authors:** Magda Zanelli, Maurizio Zizzo, Francesca Sanguedolce, Stefano Ricci, Andrea Palicelli, Alessandra Bisagni, Valentina Fragliasso, Giuseppe Broggi, Serena Salzano, Ioannis Boutas, Nektarios Koufopoulos, Ione Tamagnini, Claudia Camposeo, Andrea Morini, Rosario Caltabiano, Luca Cimino, Massimiliano Fabozzi, Paola Parente, Lucia Mangone, Alberto Cavazza, Antonino Neri, Stefano Ascani

**Affiliations:** 1 Pathology Unit, Azienda USL-IRCCS di Reggio Emilia, 42122 Reggio Emilia, Italy; stefano.ricci@ausl.re.it (S.R.); andrea.palicelli@ausl.re.it (A.P.); alessandra.bisagni@ausl.re.it (A.B.); ione.tamagnini@ausl.re.it (I.T.); claudia.camposeo@ausl.re.it (C.C.); alberto.cavazza@ausl.re.it (A.C.); 2 Surgical Oncology Unit, Azienda USL-IRCCS di Reggio Emilia, 42122 Reggio Emilia, Italy; maurizio.zizzo@ausl.re.it (M.Z.); andrea.morini@ausl.re.it (A.M.); massimiliano.fabozzi@ausl.re.it (M.F.); 3 Pathology Unit, Policlinico Riuniti, University of Foggia, 71122 Foggia, Italy; francesca.sanguedolce@unifg.it; 4 Laboratory of Translational Research, Azienda USL-IRCCS di Reggio Emilia, 42122 Reggio Emila, Italy; valentina.fragliasso@ausl.re.it; 5 Department of Medical and Surgical Sciences and Advanced Technologies “G.F. Ingrassia” Anatomic Pathology, University of Catania, 95123 Catania, Italy; giuseppe.broggi@phd.unict.it (G.B.); sere.salzano@gmail.com (S.S.); rosario.caltabiano@unict.it (R.C.); 6 Second Department of Pathology, Medical School, National and Kapodistrian University of Athens, Attikon University Hospital, 15772 Athens, Greece; iboutas@med.uoa.gr (I.B.); nkoufo@med.uoa.gr (N.K.); 7 Ocular Immunology Unit, Azienda USL-IRCCS di Reggio Emilia, 42122 Reggio Emilia, Italy; luca.cimino@ausl.re.it; 8 Department of Surgery, Medicine, Dentistry and Morphological Sciences, University of Modena and Reggio Emilia, 41124 Modena, Italy; 9 Laboratory Oncology, Fondazione IRCCS Casa Sollievo della Sofferenza San Giovanni Rotondo, 71013 San Giovanni Rotondo, Italy; p.parente@operapadrepio.it; 10 Epidemiology Unit, Azienda USL-IRCCS di Reggio Emilia, 42122 Reggio Emilia, Italy; lucia.mangone@ausl.re.it; 11 Scientific Directorate, Azienda USL-IRCCS di Reggio Emilia, 42122 Reggio Emilia, Italy; antonino.neri@ausl.re.it; 12 Pathology Unit, Azienda Ospedaliera Santa Maria di Terni, University of Perugia, 05100 Terni, Italy; s.ascani@aospterni.it

**Keywords:** plasmablastic lymphoma, primary effusion lymphoma, ALK-positive large B-cell lymphoma, HHV8-positive diffuse large B-cell lymphoma, Epstein Barr virus, Human Herpes virus 8

## Abstract

CD20-negative aggressive B-cell lymphomas are a rare and heterogeneous group of lymphomas representing a diagnostic challenge for pathologists and a therapeutic issue for clinicians, because the outcome of these patients is poor with the current therapeutic approaches. CD20-negative aggressive lymphomas include plasmablastic lymphoma, primary effusion lymphoma, ALK-positive large B-cell lymphoma and HHV8-positive diffuse large B-cell lymphoma. Conditions of immunosuppression and viral infections, such as Epstein–Barr virus and Human Herpes virus 8, are associated with all of these lymphomas with the exclusion of ALK-positive large B-cell lymphoma, which occurs in immunocompetent hosts and is not associated with viral infections. Common features of these aggressive tumors are high-grade histology with immunoblastic or plasmablastic differentiation, the absence or weak expression of mature B-cell markers such as CD20 and the frequent expression of plasma cell-associated markers. The aim of this review is to highlight the diagnostic challenges associated with the group of CD20-negative aggressive B-cell lymphomas, emphasizing key morphologic and molecular features, which are critical in the diagnosis of the different entities belonging to this rare group of diseases.

## 1. Introduction

Aggressive B-cell lymphomas, even known as large B-cell lymphomas (LBCLs), represent the most common B-cell lymphomas and are a heterogeneous group of tumors including entities with different clinical manifestations, prognoses and pathological features. Both the recently published 5th edition of the World Health Organization (WHO) classification, referred to as the WHO-HAEM5, and the International Consensus Classification (ICC) on hematological neoplasms state that the precise identification of the different entities belonging to the group of LBCLs is based on the combination of morphological, immunophenotypic and molecular features [1,2]. The accurate diagnosis is critical for patient treatment as therapy and outcome may differ among different subtypes.

The biology of most LBCLs is related to the different steps of the B-cell differentiation process in the follicle germinal center and terminal differentiation steps.

In our paper, we focus on a particular group of LBCLs typically showing downregulation of mature B-cell antigens (CD20, CD79alpha and PAX5) with the expression of plasma cell-associated markers (CD138, MUM1 and CD38). This group of LBCLs with a terminally differentiated B-cell phenotype includes lymphomas often developing in the setting of immunodeficiency and associated with Epstein–Barr virus (EBV) or Kaposi Sarcoma Herpes Virus/Human Herpes virus 8 (KSHV/HHV8) infection, such as plasmablastic lymphoma (PBL), primary effusion lymphoma (PEL) and HHV8-positive diffuse large B-cell lymphoma (HHV8+ DLBCL) [1,2]. PEL is strictly associated with KSHV/HHV8, which is the driving factor in its pathogenesis; EBV is not identified in all PEL cases, probably acting as a cofactor [3]. In PBL, EBV is detected only in a part of cases, playing a pathogenetic role in lymphomagenesis in association with other factors such as *MYC* gene rearrangement [4].

An exception within this group is represented by anaplastic lymphoma kinase-positive large B-cell lymphoma (ALK-positive LBCL), which occurs in immunocompetent individuals [1,2].

The absence of mature B-cell marker expression may render the diagnosis of this group of malignancies particularly challenging. The purpose of this review is to synthesize the current knowledge on PBL, PEL, ALK-positive LBCL and HHV8-positive DLBCL, providing pathologists with a practical guide for the diagnosis of these entities, emphasizing key morphologic and molecular features (Table 1).

## 2. Plasmablastic Lymphoma (PBL)

### 2.1. Clinical Presentation, Epidemiology and Etiology

PBL is a rare highly aggressive B-cell lymphoma, considered as a distinct entity since the 2008 WHO classification [5]. According to the WHO-HAEM5, PBL is classified as a specific subtype within the category of LBCLs [1].

The disease was originally described by Delecluse et al. in the oral cavity in association with HIV infection [6]. In addition to its well-recognized association with HIV infection, PBL may occur in individuals with other causes of immunosuppression (iatrogenic, post-transplant or immunosenescent elderly patients) and even, more rarely, in immunocompetent patients [7,8,9,10]. Rarely PBL may represent the evolution of indolent lymphomas [9].

PBL generally presents as a mass, mainly in extranodal sites, with a preference for the oral cavity and the gastrointestinal tract [1,11]. The anatomical distribution of PBL sites varies in relation with HIV status. Although lymph node involvement at disease presentation is rare, it is reported in 30% of post-transplant cases [8,9]. Advanced-stage disease at presentation is more frequent in immunosuppressed patients compared to immunocompetent individuals. Disseminated disease, even with bone marrow (BM) involvement, is reported more often in the setting of HIV-positive patients [9]. In a large cohort of cases evaluated by Castillo et al., BM involvement was present in 40% of HIV-positive patients and 25% of HIV-negative ones [9].

PBL shows a notable predominance of males, comprising up to 75% of cases; the disease may occur at any age, although it is rare in children and mainly in the HIV setting [1]. The median age at diagnosis is around 40 years in HIV-positive cases and older (around 50 years) in non-HIV patients [10].

The association of PBL with activating *MYC* rearrangements and EBV has been largely studied, as well as the genetic landscape of this aggressive lymphoma, providing an improved understanding of the disease.

EBV is detected in 60–70% of PBL cases, more often in the setting of HIV infection [9,11], and, although the pathogenesis of PBL is not completely understood, EBV certainly plays a pathogenetic role in the development of EBV-positive cases [12].

EBV is the most common persistent DNA virus, as, after primary infection, usually in childhood, the virus persists in a latent phase in B lymphocytes [7,12].

In conditions of immunosuppression, the equilibrium between virus and host immune system is impaired and, hence, EBV-associated lymphoid proliferations may develop.

EBV-associated lymphomagenesis includes the prevention of apoptosis induced by viral-encoded products and the blockage of hypomethylation in B-cells, leading to their immortalization [12,13].

### 2.2. Morphology and Immunophenotype

The disease may show a rather wide morphologic spectrum, ranging from sheets of immunoblasts (IBs) resembling diffuse large B-cell lymphoma (DLBCL) to cells with a more evident plasma cell differentiation.

**Figure 1:**
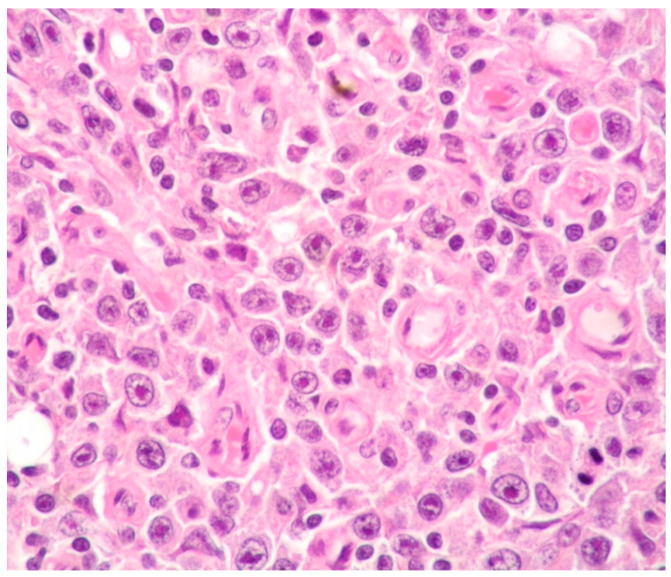
PBL: diffuse proliferation of large cells with prominent nucleoli (hematoxylin and eosin staining, magnification 400×; original image from Prof. S. Ascani).

Plasmablasts (PBs) are large cells with an eccentric large nucleus, prominent nucleolus, basophilic or amphophilic cytoplasm and often showing perinuclear clear halo-like plasma cells. Frequent mitotic figures, geographic necrosis and a starry-sky pattern may be observed in PBL (Figure 1).

The disease site and HIV status may influence PBL histology; cases with plasmablastic morphology are observed more often in HIV-positive cases of the oral mucosa, whereas cases with more mature plasmacytic features are more common in other extranodal and nodal sites [1].

**Figure 2:**
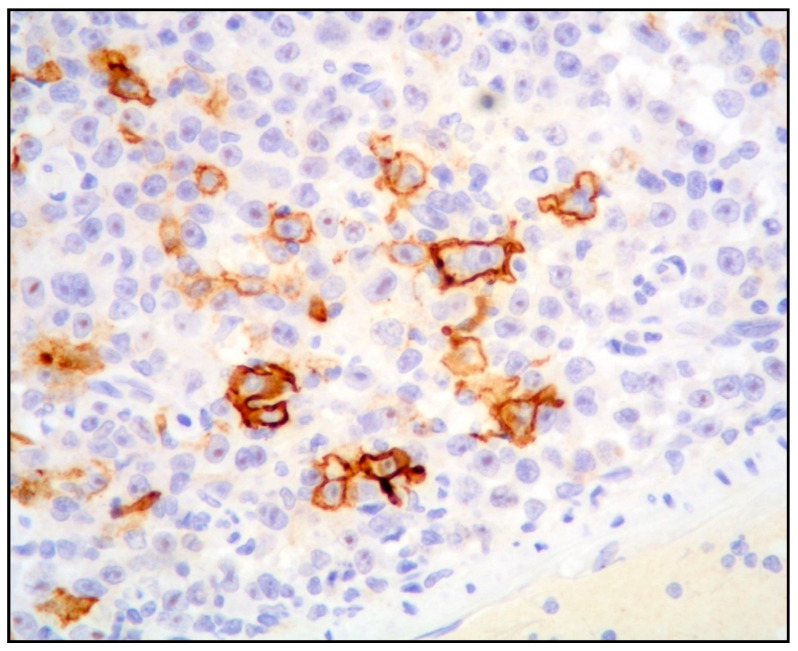
PBL: case showing expression of B-cell markers only in a minority of cells (CD20 immunostaining, magnification 400×; original image from Prof. S. Ascani).

The neoplastic cells show a terminal differentiated B-cell phenotype, with expression of plasma cell markers (CD138, CD38, MUM1/IRF4, MUM18 and PRDM1/BLIMP1) and cytoplasmic immunoglobulin light chain restriction, and negativity or weak expression of CD45 and CD79 alpha. Mature B-cell markers (CD19, CD20 and PAX5) are negative or, rarely, show a dim positivity only in a minority of cells (Figure 2).

**Figure 3:**
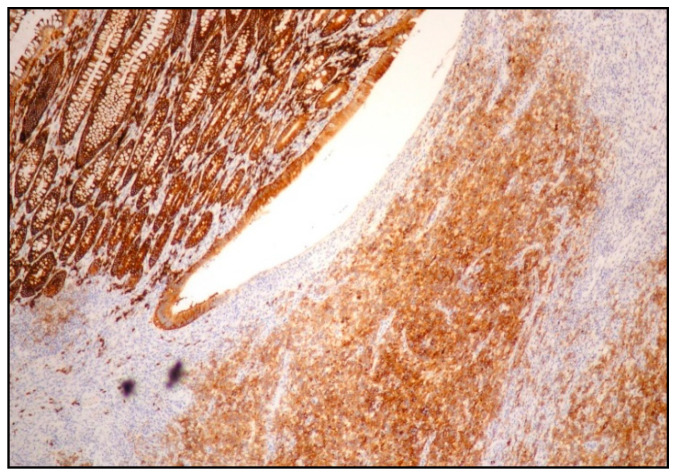
PBL: expression of plasma cell-associated markers (CD138 immunostaining, magnification 200×; original image from Prof. S. Ascani).

PBL may show an aberrant phenotype, for instance, a partial expression of plasma cell markers such as CD138 and CD38 (Figure 3).

CD56 may be found in approximately half of PBL cases, an overlapping feature with multiple myeloma (MM) [14,15]. Other immunophenotypic aberrancies include the expression of CD10, EMA and T-cell markers, in particular CD4 or CD2 [14]. The expression of CD30 and programmed cell death protein 1 (PD-1/PD-L1) is more common in EBV-positive cases, accounting for 70% of cases, and it may be relevant for potential therapies with the anti-CD30 therapy brentuximab vedotin or checkpoint inhibitors [14,16]. BCL6 and BCL2 are often negative, and HHV8 is negative by definition. The proliferative index (ki67) is elevated, usually over 80%.

MYC protein is generally overexpressed and not restricted to the *MYC*-rearranged cases, suggesting the hypothesis of alternative mechanisms of *MYC* activation and protein expression [1,2].

**Figure 4:**
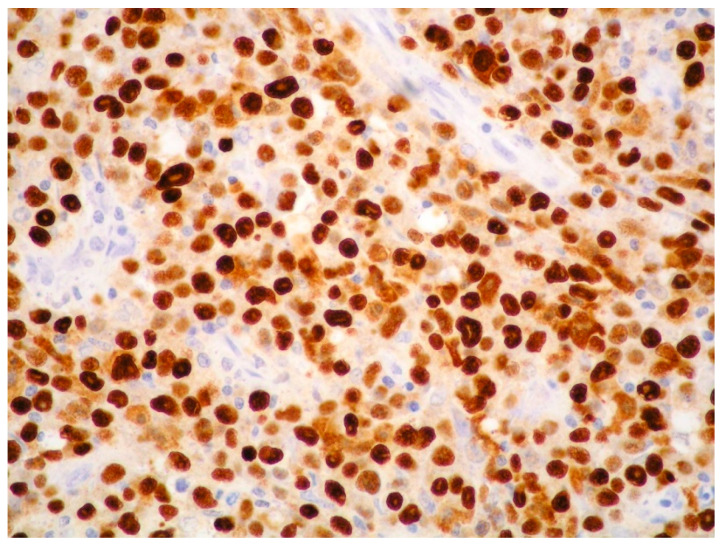
PBL: diffuse EBV expression of neoplastic cells (EBER-ISH, magnification 200×; original image from Prof. S. Ascani).

EBV infection is demonstrated by EBV-encoded ribonucleic acid in situ hybridization (EBER-ISH) positivity in tumor cells of approximately 60–75% of PBL (Figure 4).

The occurrence of EBV infection, as revealed by EBER positivity, shows a higher rate in HIV-positive (80%) and post-transplant cases (67%) compared to immunocompetent cases (50%). In EBER-positive cases, latent membrane protein 1 (LMP1) is rarely detected, indicating a type I latency pattern, which is the most frequent type of latency in PBL. Latency type II and III have been observed in HIV-positive and post-transplant PBL [11,14,17,18].

### 2.3. MYC Aberrations in PBL

*MYC* is a proto-oncogene located on chromosome 8q24, playing a critical role in the development and progression of cancer, as the MYC protein has a strong influence on biological processes such as cellular metabolism, proliferation and apoptosis [9]. *MYC* expression is normally stopped during plasma cell differentiation by transcription factors such as XPB1, IRF4 and BLIMP1 [9]. *MYC* signaling appears to be consistently activated in PBL [19].

Immunohistochemistry is used to establish the expression of the MYC protein and, in PBL, the MYC protein is usually upregulated.

The high level of the MYC protein expression in PBL is not only related to *MYC* translocation, but it is caused by several different mechanisms, as the *MYC* gene may be activated through various cancer-promoting pathways. In approximately 70% of PBL cases, MYC protein overexpression is related to *MYC* aberrations, either translocations (60%) or amplifications (<10%), whereas, in the remaining cases, MYC protein overexpression is related to *STAT3* mutations [9].

In two-thirds of PBL cases, the *MYC* gene is translocated to immunoglobulin genes, mainly the heavy chain immunoglobulin (IgH) gene on chromosome 14q35, while, in the remaining one-third, it shows translocations to non-IgH partners. *MYC* rearrangements have been seen in 50–75% of HIV-positive patients and, less frequently, in HIV-negative ones [9].

*MYC* rearrangements are usually associated with EBV positivity, CD10 expression and *PRDM1* gene mutation, in absence of mutations in the *MYC* promoter region [9,20,21]. Mutations in the *PRDM1* gene, encoding for the BLIMP1 protein and acting as an *MYC* regulator, is detected in some cases [22].

### 2.4. Other Molecular Aberrations

PBL shows an elevated genomic complexity, involving activating alterations in *JAK/STAT*, *MAPK* and *NOTCH* pathways, with frequents mutations in *MYC*, *STAT3*, *SOCS1*, *NRAS*, *TP53* and *EP300* [23,24].

The *JAK/STAT* pathway is of notable importance in regulating cellular proliferation and differentiation. Mutations affecting genes responsible for encoding elements of the *JAK/STAT* pathway have been observed in the majority (over 60%) of PBL arising in HIV-positive patients, and in more than one/third of PBL in HIV-negative ones [25]. Approximately 25% of PBL cases showed *STAT3* mutations [25]. Of note is the significant correlation between mutations in the *STAT3* gene and HIV infection; in PBL from HIV-positive patients, the occurrence of *STAT3* mutations was around 50% compared to the 10% observed in PBL from HIV-negative patients [25]. *STAT3 SH2* domain mutations causing *STAT3* activation (phospho-STAT3) represent an alternative pathway leading to MYC protein overexpression.

EBV-positive PBLs often have alterations in genes in the *JAK/STAT* pathway, and have a less complex karyotype with less *TP53* mutations and a better prognosis compared to EBV-negative PBL [23,24].

In addition to *STAT3* mutations, PBL shows mutations in other genes encoding for parts of the *JAK/STAT* pathway. A large study by Liu et al., evaluating the genomic landscape of HIV-associated PBL, showed that mutations in the *JAK/STAT* pathway were common with the *STAT3 SH2* domain, being involved in 42% of cases, *JAK1 JH1* kinase domain in 14% of cases and *SOCS1* in 10% of cases [26].

BLIMP1 is a transcription factor acting as a repressor of MYC expression in post-germinal center cells, and it is the product of the tumor suppressor gene *PRDM1*. In about 50% of PBL cases with MYC overexpression, *PRMD1* missense mutations involving domains essential for the regulation of *MYC* gene expression are identified [22,27]. MYC protein overexpression, caused by either *MYC* alterations or *STAT3* mutations, and *PRMD1* mutations are more common in EBV-positive PBL and may be favored by EBV-related genomic instability [27,28].

Recurrent somatic copy number alterations (CNAs) have also been identified in PBL, mainly amplifications involving chromosomes 1q, 7p and 7q [24,25]. Of note for potential therapeutic implications, the amplification of chromosome 1q21.3, including the anti-apoptotic protein MCL1, which is the target of MCL1 inhibitors [24,29].

### 2.5. Treatment

PBL is an aggressive lymphoma with a median overall survival (OS) of 3 months if untreated; however, the outcome of PBL remains poor with current therapeutic strategies [1]. Chemotherapy represents the main treatment of PBL. Although CHOP regimen (cyclophosphamide, doxorubicin hydrochloride, vincristine sulfate and prednisone) is the most common treatment choice in poor-resource countries, the NCCN guideline recommends more intensive chemotherapeutic protocols, such as dose-modified (DA)-EPOCH (etoposide, prednisone, vincristine, doxorubicin and cyclophosphamide), CODOX-M/IVAC (cyclophosphamide, vincristine, doxorubicin and high-dose methotrexate alternated with ifofosfamide, etoposide and high-dose cytarabine) or Hyper-CVAD (cyclophosphamide, vincristine, doxorubicin and dexamethasone alternated with cytarabine and high-dose methotrexate) [30]. However, the treatment efficacy of more intensive chemotherapeutic regimens still remains controversial, with several studies showing no significant difference in survival between patients receiving CHOP and patients treated with more intensive regimens [31,32]. Chemotherapeutic regimens for plasma cell tumors, such as the proteasome-inhibitor Bortezomib alone or combined with chemotherapy, may obtain good results, but large randomized studies are needed [33,34].

The use of immune modulators such as lenalidomide is reported in very few studies; however, although a large number of studies is lacking, lenalidomide alone or combined with other treatments may be of help in maintaining long-term clinical remission in PBL patients [35].

In PBL, the PD-1/PD-L1 pathway is abnormally activated, with a high expression of PD-1 and PD-L1 in particularly in EBV-positive cases [36].

The interaction between PD-L1 expressed on tumor cells with PD-1 on the T-cell surface is responsible of the inhibition of T-cell-mediated immune response, and causes tumor cell escape from antitumor immune surveillance [37,38,39]. The immunotherapy is based on the use of antibodies, called immune check point inhibitors (ICIs), blocking the PD-1/PD-L1 pathway. The blockade of the PD-1/PD-L1 axis by ICIs releases T-cells from the inhibitory effect of tumor cells, re-establishing T-cell antitumor function and causing tumor cell elimination [38,39,40,41]. The therapeutic role of ICIs in PBL is described in a very limited number of papers and needs further investigation [42].

Chimeric antigen receptor T-cell (CAR-T) is a recently introduced immunotherapy using T-cells engineered with synthetic receptors. CAR-T therapy has an antitumor effect, because CAR-T cells recognize and eliminate cancer cells [43]. In the near future, CAR-T therapy might represent a treatment option for PBL patients [44].

The efficacy of autologous hematopoietic stem cell transplantation (ASCT) in PBL patients needs further large studies, as the data obtained so far are based on small case series [45].

Chemotherapy followed by radiation therapy consolidation may improve survival in limited-stage PBL cases [46].

In HIV-positive PBL patients, the prognosis remains poor despite the use of intensive chemotherapy associated with highly active antiretroviral therapy (HAART) [47].

There is an urgent need to develop more effective treatments in this aggressive and difficult-to-treat malignancy.

## 3. Primary Effusion Lymphoma (PEL)

### 3.1. Clinical Presentation, Epidemiology and Etiology

PEL is a distinct HHV8-positive highly aggressive B-cell lymphoma. Its distinctive feature is the occurrence within large body cavities, such as the peritoneal, pericardial and pleural cavities, usually without a detectable tumor mass, although the spreading of lymphoma from body cavities to contiguous tissues may be observed [1,2,48,49,50,51]. PEL patients generally have symptoms derived from fluid accumulation, such as dyspnea, chest pain and abdominal distension.

Lymphomas with the same pathological features as PEL can present as solid tumor masses outside body cavities, and are labeled as extracavitary PEL (EC-PEL) in both the WHO and ICC classifications [1,2,52,53,54,55,56,57,58,59,60,61]. EC-PEL, which accounts for approximately 5% of all PEL cases, more often involves lymph nodes as a solitary tumor mass and, more infrequently, extra-nodal sites such as the gastrointestinal tract (GIT), skin, lung and central nervous system (CNS) [52,53,54,55,56,57,58,59,60,61]. In EC-PEL, body cavity involvement may develop during the course of the disease [60].

KSHV/HHV8 is the etiologic agent of PEL. A selection of HHV8 viral genes is critical for PEL development; therefore, HHV8 is considered the genetic driver of PEL, and HHV8 identification by immunohistochemistry is a required criterion for PEL diagnosis [1,2].

PEL, either the classic form and the EC variant, affects a rather unique population of individuals who are generally immunocompromised or elderly.

Similarly to Kaposi sarcoma (KS), in which HHV8 was first identified, different epidemiological subtypes of PEL have been observed [1,2,62].

The prevalent variant of PEL is the one occurring in HIV-infected individuals; HIV-associated cases develop more often in young male individuals, present an aggressive clinical course and are usually coinfected by HHV8 and EBV [1,2,48,49,50].

The Mediterranean variant of PEL develops in HIV-negative elderly individuals, usually from geographic areas where HHV8 infection is endemic, and in the absence of other causes of immunodeficiency besides immunosenescence [1,2]. In this variant, EBV is generally absent and the clinical course is more indolent [1,2,63].

PEL may also develop in another setting of immunosuppression, like post-transplantation, more often in solid organ recipients [1,2,51,64,65,66].

Post-transplant (PT) PEL generally has the classical PEL presentation with body cavity effusions and rarely presents in its EC variant [59]. EBV coinfection is usually absent in PT-PEL [51].

### 3.2. KSHV/HHV8

While EBV is a ubiquitous virus, infecting the majority of people worldwide, KSHV/HHV8, a member of the gamma-herpesvirus family, is endemic in specific geographic areas, such as the Mediterranean region, Caribbean, Latin America, sub-Saharan Africa and Middle Eastern countries [1,2]. HHV8 may be transmitted through saliva, but it may be also transmitted sexually, through blood and vertically through breast milk [48,60].

HHV8 has the ability to infect various cell types, including lymphocytes, and, similarly to EBV, can persist lifelong in a latent form in lymphocytes. In conditions of immunodeficiency, when the balance between the virus and the host immune system is disrupted, HHV8 may reactivate the lytic replicative cycle producing viremia [48].

HHV8 may lead to the development of different diseases in general in patients with compromised immune systems. The virus was first identified in KS and named KS-herpesvirus (KSHV) [62]. Subsequently, HHV8 was identified to be the causative agent of HHV8-positive multicentric Castleman’s disease (HHV8-positive MCD) [67] and PEL [48].

HHV8 is also associated to HHV8-positive DLBCL, an aggressive LBCL mainly occurring in MCD patients [1,2]. More recently, HHV8 has been identified to be associated with germinotropic lymphoproliferative disorder (GLPD), which is the only HHV8-related disease developing in immunocompetent hosts [1,2,68,69,70].

As mentioned above, PEL more often develops in the context of immunodeficiency and, interestingly, even in transplanted individuals [51,59]. The long-term immunosuppression is the predisposing factor for PT-PEL, which usually develops several years after transplantation, unlike EBV-associated post-transplant lymphoproliferative disorders (PTLDs), which are more frequent in the early post-transplant phase.

PT-PEL may develop because a pre-existing HHV8 infection in the recipient host is reactivated or because HHV8 infection is acquired by an HHV8-negative recipient of an organ from an infected donor. The reactivation of a pre-existing HHV8 infection is considered the most common way in organ recipients from countries with high HHV8 serological prevalence [64,65,66].

### 3.3. Morphology, Immunophenotype and Molecular Features

**Figure 5:**
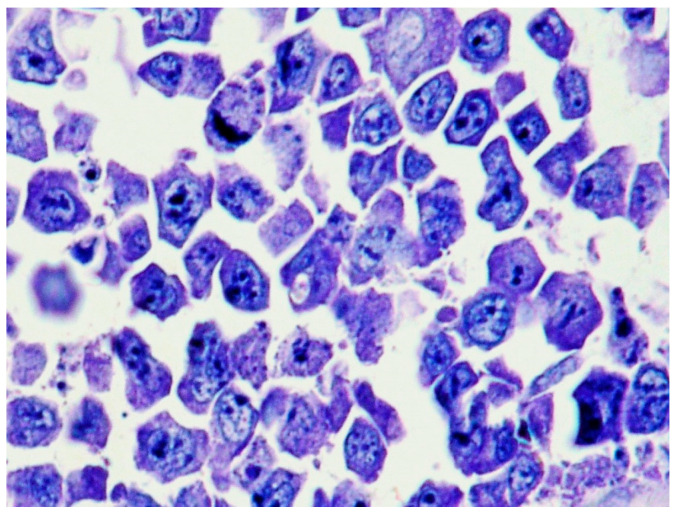
EC-PEL: high-power view showing the cytological features of large neoplastic cells with evident nucleoli (Giemsa staining, magnification 400×; original image from Prof. S. Ascani).

Both classic PEL and EC-PEL show the same morphological features, with large cells varying in appearance from IBs to PBs and anaplastic cells (Figure 5).

The neoplastic cells are classically positive for plasma cell markers, such as CD138, MUM18 and MUM1/IRF4, and markers of lymphoid activation, such as CD30, CD38, EMA and HLA-DR. The aberrant expression of T-cell markers is reported with a higher frequency in EC-PEL.

Being a LBCL showing downregulation of mature B-cell antigens, PEL, at least in its classic cavitary form, usually does not express classic B-cell markers (CD19, CD20, CD79α and PAX5) and surface and cytoplasmic immunoglobulin. The classic form is usually positive for CD45/LCA. EC-PEL shows some immunohistochemical differences compared to the classic cavitary form. EC-PEL may express B-cell markers with a higher frequency (25%) compared to the cavitary form (5%), as well as immunoglobulin (25% vs. 15%), and it expresses CD45 with a lower frequency compared to classic PEL [54].

**Figure 6:**
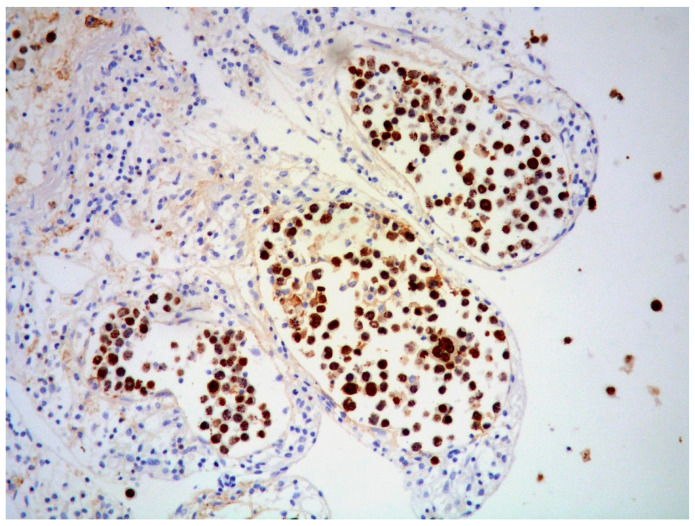
EC-PEL: diffuse expression of HHV8 in the neoplastic cells (HHV8 immunostaining, magnification 200×; original image from Prof. S. Ascani).

The expression of HHV8 is a required criterion for PEL diagnosis. Using immunohistochemistry, the expression of HHV8-encoded latency-associated nuclear antigen 1 (LANA-1) protein is detected (Figure 6).

EBV is identified by EBER in approximately 70% of PEL cases, and EBV coinfection is usually present in the setting of HIV infection. EBV-negative PELs usually occur in HIV-negative cases and especially in elderly patients from HHV8 endemic zones. EBV-latent membrane protein 1 (LMP1) is usually negative, indicating a type I latency pattern, similar to PBL. Clonality for the immunoglobulin gene is usually present, although even T-cell receptor (TCR) gene rearrangement may sometimes be identified. HHV8 viral genomes are identified. Despite the presence of MYC protein overexpression, likely caused by HHV8-encoded latent proteins, no abnormalities in the *MYC* gene are identified, as well as no rearrangements in *BCL2* and *BCL6* loci [14]. HHV8-encoded latent proteins play a relevant oncogenic role in PEL. These proteins are linked to the inhibition of apoptosis (*TP53* and *RB* genes) and the activation of the *NF-kB*, *JAK/STAT* and *PI3K/AKT/mTOR* pathways [14]. Hence, HHV8 represents the driving force in PEL; however, EBV may be a potential cofactor in the development and proliferation of EBV-positive cases [71]. Xu et al. reported a possible role of EBV in favoring the establishment of HHV8 by inhibiting its lytic replication [72].

### 3.4. Outcome and Treatment

The clinical course of PEL is very aggressive, with an overall survival rate of less than 12 months despite the use of intensive chemotherapeutic regimes [1,2].

Apparently, in the setting of HIV, classic PEL shows a worse behavior compared to EC-PEL, with a median survival of 3 months vs. 11 months [52]. Rituximab is not generally used due to the lack of CD20 expression, at least in classic PEL.

In HIV-positive cases, PEL is treated with systemic chemotherapy (often a CHOP-based regimen) along with highly active antiretroviral therapy (HAART) [52].

However, CHOP-based chemotherapy has scarce efficacy in PEL, and more intensive chemotherapeutic protocols, such as EPOCH, or treatments targeting the proteasome, the NF-kB pathway or anti-CD30 therapy are required [14,73].

In PT-PEL, chemotherapy is associated, if possible, with the reduction in immunosuppressive treatment and antivirals (particularly in case of high viral load) [65]. In case of chemotherapy failure, antiviral drugs such as cidofovir, either systemic or intracavitary, are taken into consideration as salvage therapy [65].

## 4. ALK-Positive Large B-Cell Lymphoma (ALK-Positive LBCL)

### 4.1. Clinical Presentation, Epidemiology and Etiology

ALK-positive LBCL is a CD20-negative, aggressive LBCL first described by Delsol et al. in 1997 [74]. It is rare, representing less than 1% of all LBCLs, and, since the Delsol description, it has been reported in fewer than 200 cases, possibly because it is underrecognized [75,76,77,78,79,80,81,82,83,84,85,86,87,88,89,90,91,92,93,94,95,96,97,98]. In the WHO-HAEM5, ALK-positive LBCL is still classified as a specific subtype within the category of LBCLs [1].

Due to its rarity, data on ALK+ LBCL are rather scarce, and limited to single reports or small case series [74,75,76,77,78,79,80,81,82,83,84,85,86,87,88,89,90,91,92,93,94,95,96,97,98].

The disease can affect individuals of any age (range 9–90), with a median age of around 40 years and a male prevalence (M/F = 4:2). The clinical course is aggressive, and patients often present with high-stage disease and a median survival of 11–24 months. Nodal clinical presentation is more common (56%) than unique extranodal disease (14%), whereas one-third of cases show concomitant nodal and extranodal involvement [77]. Similarly to PBL and PEL, it shows high-grade histological features and plasmablastic differentiation, and it is negative for mature B-cell markers. Unlike PBL and PEL, there is no association with immunodeficiency or infections such as EBV and HHV8.

### 4.2. Morphology, Immunophenotype and Molecular Features

The disease often involves lymph nodes with a sinusoidal and/or diffuse growth pattern. In a recent review by Castillo et al., cases at early stages were noted to be predominantly sinusoidal [77]. Areas of necrosis are often present. Neoplastic cells are large with immunoblastic and/or plasmablastic morphology.

Similarly to other neoplasms with plasmablastic differentiation, ALK+ LBCLs express plasma cell-associated markers (CD138, CD38, MUM1/IRF4 and BLIMP1) and are negative or only weakly positive for mature B-cell markers (CD20, CD19, PAX5 and CD79alpha). Some cases are positive for B-cell transcription factors (OCT2 and BOB1), and the expression of the monotypic immunoglobulin light chain is found in about 85% of cases. The aberrant expression of the T-cell marker CD4 and the NK-cell marker CD57 is found in approximately half of cases. CD30 is usually negative. cMYC may be expressed, and CD45 is found in about 70% of cases.

The incorporation of ALK staining in the histopathological characterization of all cases of LBCLs, in particular those with an immunoblastic/plasmablastic morphology, is essential in identifying ALK-positive LBCL; otherwise, the disease remains underrecognized.

Diagnosing ALK-positive LBCL can be challenging. The loss of B-markers, along with the possible lack of CD45 and the variable expression of epithelial markers (cytokeratin AE1/AE3 and EMA), can lead to the misdiagnosis of this lymphoma as metastatic carcinoma.

The immunostaining pattern of ALK may vary according to different fusion partners of ALK. The staining of ALK may present as a nuclear and cytoplasmic staining pattern, or it may be restricted to the cytoplasm with a diffuse or granular pattern. The most prevalent cytogenetic alteration is the t(2;17)(p23;q23), involving the clathrin gene (CLTC) at chromosome band 17q23 and the ALK gene at chromosome band 2p23 [78]. Although rare, other *ALK* rearrangements have been identified, such as *NPM1* [79,80], *SEC31A* [80], *SQSTM1* [81], *RANBP2* [82], *IGL* [83], *EML4* [84], *GORASP2* [85], *TFG* [86] and *PABPC1* [87].

With the exception of cases with *NPM1::ALK* fusion, which show both nuclear and cytoplasmic staining, the other genetic alterations display cytoplasmic ALK staining. In detail, cases with *CLTC::ALK* fusion show a characteristic cytoplasmic granular staining pattern, concentrated in the perinuclear Golgi zone; cases with *SEC31A::ALK* fusion, as well as cases with *PABPC1::ALK* fusion, display a diffuse cytoplasmic staining pattern, and cases with *TFG::ALK* fusion show a non-uniform cytoplasmic granular staining [88]. Most patients have IgH gene clonal rearrangement, and *MYC* rearrangement is absent.

### 4.3. Outcome and Treatment

ALK-positive LBCL is an aggressive disease; most cases are diagnosed at an advanced stage and the prognosis is unfavorable, with a median survival of less than 24 months. Of note, the prognosis depends largely on the clinical stage at presentation. Moreover, patients younger than 35 years seem to have a better prognosis compared to patients older than 35 [75,89].

Treating this disease is a considerable challenge, often being a chemotherapy and radiotherapy refractory disease with a poor prognosis. There is no standardized treatment for this lymphoma. The lack of B-cell markers, such as CD20 and CD19, renders ineffective anti-CD20 antibodies, such as rituximab, as well as the CD19-directed CART therapy. Most patients are treated with chemotherapy receiving CHOP, CHOP-like regimens or more intensive protocols, and some underwent localized radiotherapy and HSCT. When treated with the conventional CHOP protocol, the disease usually follows a worse outcome compared with DLBCL, NOS, with a 5-year overall survival of only 25% [75,89]. ALK-positive LBCL can be cured with CHOP protocols only in cases of localized disease [75,89]. HSCT may be recommended for patients with advanced stage disease [76]. Due to the scarce results with conventional therapies, the use of new drugs, such as ALK inhibitors, is of particular importance. ALK inhibitors are effective in the treatment of inflammatory myofibroblastic tumor, non-small-cell lung cancer with ALK rearrangement and ALK-positive ALCL [90,91], and represent a therapeutic option for ALK-positive LBCL [92,93]. Crizotinib is a first-generation ALK inhibitor which is effective in ALK-positive LBCL, but the duration of response may be short; potent next-generation ALK-inhibitors such as alectinib and lorlatinib showed promising results [94,95,96]. In addition, ALK expression in this disease is linked to STAT3 phosphorylation and, hence, STAT3 may represent one of the molecular targets for this lymphoma [97,98].

## 5. HHV8-Positive Diffuse Large B-Cell Lymphoma (HHV8-Positive DLBCL)

### 5.1. Clinical Presentation, Epidemiology and Etiology

HHV8-positive DLBCL was previously known as LBCL arising in HHV8-associated MCD in both the 2008 and 2017 WHO classifications [5,99]. It is a rare and aggressive subtype of LBCL, usually linked to HHV8-positive MCD and usually observed in patients with HIV, although it may arise even in other conditions of immunosuppression [1,2,100,101,102,103,104,105,106,107,108,109,110,111,112].

The disease generally affects adults with a median age of 47 years and a male prevalence (M/F = 3.3:1), and characteristically involves multiple lymph nodes and the spleen with massive splenomegaly; extranodal sites may be involved, as well as BM with peripheral blood involvement [105].

### 5.2. Morphology, Immunophenotype and Molecular Features

The development of lymphoma is characterized by confluent sheets of large cells with a plasmablastic or immunoblastic appearance, with the effacement of the normal architecture of lymph nodes, spleen or extranodal sites. The neoplastic cells strongly express cytoplasmic IgM, often with lambda light chain restriction (more unfrequently, it can express kappa) [102]. The tumor cells are variably positive for CD20 and CD45, and express terminal B-cell differentiation markers such as MUM1, whereas they are negative for CD79alpha, PAX5, CD138, CD30, ALK, CD3 and EBER. HHV8 positivity is required for diagnosis [102]. HHV8-positive DLBCL arising in HHV8-positive MCD originates from PBs, which are present either as single cells or as peri- and intrafollicular small clusters in MCD. PBs in HHV8-positive MCD show the same phenotype of HHV8-positive DLBCL. In some cases, the PBs of HHV8-positive MCD may continue to grow in large aggregates until destroying the normal tissue architecture and meeting the diagnostic criteria for lymphoma. The term microlymphoma, indicating small aggregates of PBs, is not currently used, as these aggregates are not clonal diseases and do not constantly progress to frank lymphoma.

In MCD, the individual PBs are polyclonal, whereas the clusters may be polyclonal or oligoclonal. In overt lymphoma, a monoclonal immunoglobulin gene rearrangement is present, and molecular studies demonstrated that HHV8-positive DLBCL is made up of monoclonal B-cells without somatic hypermutation of IG genes and, hence, consistent with naïve B-cells [100,102,104].

### 5.3. Outcome and Treatment

The prognosis of HHV8-positive DLBCL is poor, ranging from weeks to months, and there are no standard treatment protocols. The outcome of this lymphoma is very different from HHV8-negative DLBCL arising in HIV-positive individuals, where over 75% of patients treated with intensive chemotherapy are alive at 3 years [106]. Due to the rarity of the disease and its poor response to conventional chemotherapy with the CHOP regimen, HHV8-positive DLBCL remains a therapeutic challenge [109]. Treatment strategies often include intensive chemotherapeutic protocols, such as CODOX-M/IVAC or DA-EPOCH, and autologous or allogenic stem cell transplantation [110]. Novel therapeutic strategies targeting HHV8, including antiviral treatments, immunomodulary therapies and small molecular inhibitors, are under evaluation, although large studies are necessary to define their role in the treatment of this aggressive lymphoma [111,112].

### 5.4. Summary of the Main Differential Diagnoses in Cases of Lymphomas with Plasma Cell Differentiation

The differential diagnosis of lymphomas sharing features of plasma cell differentiation should take into consideration aggressive diseases such as PBL, plasmablastic myeloma (PM), PEL, EC-PEL, ALK-positive LBCL, Burkitt lymphoma (BL), HHV8-positive DLBCL and EBV-positive DLBCL, NOS [1,2].

The differential diagnosis between PBL and PM can be challenging, as these malignancies have similar morphological and immunophenotypic features.

Due to overlapping characteristics between PM and PBL, a strict correlation with clinical, radiological and laboratory findings is necessary to distinguish these entities.

The diagnosis of PBL is favored when there is an underlying condition of immunosuppression; the association with EBV tends to support the diagnosis of PBL as well.

PM represents a rare morphological subtype of plasma cell myeloma, and patients with PM generally have a significant serum monoclonal immunoglobulin, which is not common in PBL. The presence of lytic bone lesions, renal dysfunction, monoclonal paraproteinemia, hypercalcemia and urine light chain proteins are more in favor of PM.

However, lytic bone lesions may be rarely observed even in PBL, and both PM and PBL may have bone marrow involvement.

From the histological point of view, in the differential diagnosis between PBL and PM, detecting EBER may be considered diagnostic of PBL; in contrast, the expression of cyclin D1 and CD117 supports PM.

Having said that, a definitive distinction may be difficult in some cases, and the current WHO classification suggests to use the term of “plasmablastic neoplasm consistent with PBL or PM” [1].

PEL as well as PBL arises in the context of immunosuppression. The classic form of PEL is usually limited to body cavities, whereas its EC variant more often involves nodal sites, unlike PBL, which is more often an extranodal disease. PEL is a terminally differentiated LBCL and, therefore, similarly to PBL, is positive for plasma cell-associated markers, EMA and CD30, and negative for mature B-cell markers, although B-cell markers may more often be found in the solid/EC variant of PEL.

Immunoglobulin expression is usually absent in PEL, unlike in PBL; however, immunoglobulin expression may be detected in the EC variant of PEL. HHV8 is by definition positive in PEL and negative in PBL. EBV expression may be detected in PEL, usually in HIV-positive cases.

Unlike PBL and EC-PEL, ALK-positive LBCL is an aggressive lymphoma occurring in immunocompetent individuals. ALK is a crucial marker to apply to all LBCLs with an immunoblastic/plasmablastic appearance. Unlike PBL, ALK-positive LBCL is negative or dimly positive for CD30 and is always EBV-negative.

ALK-positive anaplastic large cell lymphoma (ALCL) needs to be considered in the differential diagnosis with ALK-positive LBCL due to morphology, CD20 negativity and ALK expression. ALK-positive ALCL is a lymphoma with a T-cell/null phenotype, characterized by *NPM/ALK* fusion and usually following a good prognosis. CD30 is a crucial marker in distinguishing ALK-positive ALCL, typically showing diffuse CD30 expression from ALK-positive LBCL, which is CD30-negative or only weakly positive [88].

The differential diagnosis between EC-PEL and HHV8-positive DLBCL may be difficult. An underlying HHV8-positive MCD, multiple lymph node involvement, expression of IgM and negativity for CD138 and CD30 are more suggestive of HHV8-positive DLBCL. It has to be mentioned that patients with HHV8-positive MCD may develop PEL; however, unlike in HHV8-positive DLBCL, there is no evidence of progression from HHV8-positive MCD to PEL [54,104].

As already mentioned in the differential diagnosis of lymphomas with plasma cell differentiation, it is essential to exclude even BL and EBV-positive DLBCL, NOS, which, however, express mature B-cell markers, and their discussion goes beyond the scope of the present review.

Similarly to PBL, BL often occurs in HIV-positive individuals and, in some cases, it may show features of plasma cell differentiation, as well as association with EBV and *MYC* rearrangement. Unlike PBL, BL is a germinal-center-derived lymphoma, expressing mature B-cell markers, such as CD20, in association with germinal center markers (CD10 and BCL6).

Unlike other lymphomas with plasma cell differentiation, EBV-positive DLBCL, NOS is negative for plasma cell-associated markers and strongly express mature B-cell markers (CD20 and PAX5), more often with a non-germinal (activated) phenotype [1,2].

## Figures and Tables

**Table 1:** Main differential diagnoses of CD20-negative aggressive B-cell lymphomas.

	PEL	PBL	ALK+ LBCL	HHV8+ DLBCL
**HIV**	+ (− in elderly)	+ (often)	-	Generally +
**Immunodeficiency**	Usually present (HIV-associated, post-transplant, immunosenescence)	Usually present (HIV-related, post- transplant or iatrogenic)	-	Usually present
**Age/Sex/** **Outcome**	Adults, often males (HIV+ patients are younger) poor	Often adults, frequently males (HIV+ patients are younger) poor	Adults, often males poor	Adults poor
**Clinical manifestations**	Classic PEL: body cavities effusion EC-PEL: lymph nodes and extranodal sites	More often extranodal disease; rarely lymph nodes	More often nodal disease (in 1/3 of cases, nodal and extranodal disease)	Multiple lymph nodes, extranodal sites, spleen, BM
**Association with HHV8+ MCD**	rare	-	-	frequent
**Histology**	Large cells with IB and PB features	Diffuse proliferation of PBs/IBs	Sinusoidal and/or diffuse growth pattern of IBs and PBs	Sheets of PBs/IBs effacing organ architecture
**CD20**	- (may be + in EC-PEL)	- or weakly + in a small number of cells	- (or weakly +)	+/−
**PAX5**	- (may be + in EC-PEL)	- or weakly + in a small number of cells	- (or weakly +)	-
**CD79alpha**	- (may be + in EC-PEL)	+ in 40% of cases	- (or weakly +)	-
**MUM1/IRF4**	+	+	+	+
**CD38**	+	+	+	−/+
**CD138**	+	+	+	-
**CD30**	+	+	-	- (rarely +)
**EMA**	Often +	+	+	-
**ALK**	-	-	+	-
**T-cell markers**	Occasionally + (mainly in EC-PEL)	Occasionally +	+ in half of cases (CD4)	-
**Light chain restriction**	Usually absent	+ (often IgG kappa or lambda)	+ (in 85% of cases)	+ cIgM lambda
**HHV8**	+	-	-	+
**EBV (by EBER)**	+ (EBER− in HIV-negative elderly individuals and in post transplantation cases)	+ in 60–75% of cases (more often in HIV+ and post-transplant cases)	-	-
**Clonality**	Monoclonal (IG genes hypermutated).	Monoclonal	Monoclonal	Monoclonal (IgG genes unmutated)

**Legends**: ALK+ LBCL: ALK-positive large B-cell lymphoma; BM: bone marrow; EBV: Epstein–Barr virus; EBER: in situ hybridization for EBV-encoded RNA; EC: extracavitary; EC-PEL; extracavitary primary effusion lymphoma; HHV8+ MCD: HHV8-positive multicentric Castleman disease; HHV8+ DLBCL: HHV8-positive diffuse large B-cell lymphoma; IBs: immunoblasts; PBs: plasmablasts; PBL: plasmablastic lymphoma; PEL: primary effusion lymphoma.

## Data Availability

Individual patient data from the original studies included in the present review are not available and data sharing at this level is not applicable for a review.
